# FGF-23, Klotho and Vitamin D Levels in Scleroderma

**Published:** 2017-04

**Authors:** Ravan AHMADI, Mehrzad HAJIALILO, Amir GHORBANIHAGHJO, Ali MOTA, Sina RAEISI, Nasrin BARGAHI, Mohammad VALILO, Farahnaz ASKARIAN

**Affiliations:** 1. Dept. of Clinical Biochemistry and Laboratory Medicine, Tabriz University of Medical Sciences, Tabriz, Iran; 2. Biotechnology Research Center, Tabriz University of Medical Sciences, Tabriz, Iran

**Keywords:** FGF-23, Klotho, Scleroderma, 25-hydroxy vitamin D

## Abstract

**Background:**

Scleroderma is a chronic connective tissue disease of unknown etiology. Vitamin D and parathyroid hormone (PTH) that play particular functions in calcium and phosphate homeostasis may be involved in the etiology of this disorder. Klotho, the co-receptor of the fibroblast growth factor 23 (FGF-23), can interfere with calcium and phosphate metabolism. The purpose of this study was to evaluate serum Klotho, FGF-23, intact PTH (iPTH) and vitamin D levels in scleroderma patients compared with the healthy controls.

**Methods:**

The study was performed in Biotechnology Research Center, Tabriz University of Medical Sciences (TUMS) from 2014–2015. Sixty scleroderma patients based on the classification criteria of systemic sclerosis and 30 age- and sex-matched healthy controls were included in this study. Serum Klotho, FGF-23, 25-hydroxy vitamin D (25-OH Vit D), and iPTH levels were analyzed using ELISA.

**Results:**

Serum levels of Klotho and 25-OH Vit D in the scleroderma patients were lower than those in the healthy controls (*P*<0.001). In addition, scleroderma patients had higher serum iPTH levels than the controls (*P*<0.001). There was no significant difference in serum FGF-23 levels between the patients and controls (*P*=0.202).

**Conclusion:**

The decreased serum Klotho, 25-OH Vit D, and increased iPTH levels in the scleroderma patients may be associated with the pathogenesis of this disease and could be considered a future therapeutic target.

## Introduction

Scleroderma or systemic sclerosis is a chronic connective tissue disease. This disorder is one of the autoimmune rheumatic diseases with a variable clinical course (1). The estimated incidence of systemic sclerosis in the United States is 20 cases per million populations and its prevalence has been estimated as 276 cases per million populations; however, the reported prevalence varies depending on the used methodology and the targeted population. Systemic sclerosis occurs worldwide, but its reported prevalence varies significantly in different countries. It affects adults and children but is most common in women aging 30 to 50 (1, 2).

There are three main features in pathogenesis: 1) production of autoantibodies, 2) a non-inflammatory vasculopathy, and 3) fibroblast dysfunction leading to the increased deposition of extracellular matrix and its accumulation in skin and internal organs. Hardening of the skin is one of the most observable manifestations of this disease. Scleroderma has two different subgroups: “limited scleroderma” is so named because skin involvement is limited to the hands and face. This form is characterized by calcinosis, Raynaud’s phenomenon, esophageal dysmotility, sclerodactyly, and telangiectasia (CREST syndrome). Another form of disease is “diffuse scleroderma” that appears with the wider extent of skin involvement and internal organs such as the lungs, gastrointestinal tract, heart, and kidneys (3, 4).

Scleroderma has a complex etiology and multifactorial causes such as genetic predisposition, environmental factors, and hormonal effects (3, 5). Recently, the role of vitamin D as a potent environmental factor modulator of autoimmune diseases has been verified (6). This vitamin acts as a steroid hormone with an intracellular receptor that has multiple regulatory and functional effects throughout the body. The dominant role of vitamin D is in calcium homeostasis. Vitamin D deficiency has been recognized at high percentage of scleroderma patients and its level can be related to the severity of the disease (7).

Klotho is a single-pass transmembrane protein with a large extracellular domain and a short intracellular portion. Klotho predominantly is expressed in the renal tubules and, then, in the parathyroid glands and choroid plexus of the brain (8). There are two forms of Klotho: membrane-bound Klotho that acts as a co-receptor for the fibroblast growth factor-23 (FGF-23) and soluble Klotho(s-Klotho) is derived by alternative splicing or cleavage from membrane-Klotho. No roles of this protein have been completely understood yet thus far; however, it can get involved in calcium and phosphate homeostasis (9, 10). FGF-23 is a peptide released from bone tissue osteocytes and osteoblasts (11). It plays an important role in the bone–kidney axis and the regulation of calcium and phosphate homeostasis. Klotho as a co-receptor is necessary for FGF-23 activities (9, 10).

Calcifications of skin, vascular, and internal organs are important clinical findings in the subjects affected by scleroderma. According to the important roles of vitamin D, FGF-23, as well as Klotho in calcium metabolism, the aim of the present study was to evaluate serum Klotho, FGF-23, and 25-hydroxy Vit D levels in the scleroderma patients compared with the healthy controls.

## Materials and Methods

### Patient selection

The study was performed in Biotechnology Research Center, Tabriz University of Medical Sciences from 2014–2015.

First, The Ethics Committee of the university approved the study. After obtaining informed consent from the patients, 60 scleroderma patients (30 with diffuse scleroderma and 30 with limited scleroderma) based on 2013 classification criteria for systemic sclerosis were recruited (12) and also 30 age- and sex-matched normal subjects were included in the study. Recruitment of the patients occurred in Sheikh Al-Raeis and Atieh Rheumatology Clinics from May 2014 through June 2015. Screening, demographic measurements, and clinical assessment were performed by the rheumatologists involved in this research protocol. The Medsger severity scale (13) was considered for the patients. The patients with overlapping syndrome, impaired renal function, liver disease, taking vitamin D (patients must stop taking vitamin D at least two months prior to the inclusion), various malignancies, metabolic disorders, and abnormal parathyroid function were excluded from the study. In all the cases, a blood sample was taken in the morning after an overnight fasting and stored at −70 °C until the assays.

The sera were tested for creatinine, urea, calcium, phosphorus, alkaline phosphatase (ALP), Aspartate transaminase (AST), Alanine transaminase (ALT), intact parathyroid hormone (iPTH), 25-hydroxy vitamin D (25-OH D), FGF-23, and Klotho. Calcium, phosphorus, creatinine, AST, ALT, and ALP were determined colorimetrically using commercial reagents in an automated chemical analyzer (Roche Cobas Mira). The iPTH was measured by enzyme-linked immunosorbent assay (ELISA) (Immunodiagnostic Systems, Boldon, UK). Serum Klotho (Eastbiofarm, China), 25-Hydroxy vitamin D [25-(OH)-D] (Immunodiagnostic Systems, Boldon, UK), and FGF-23 (Eastbiofarm, China) were measured by ELISA according to the manufacturer’s recommendations.

### Statistical analysis

Data were analyzed by statistical software SPSS 18 (Chicago, IL, USA) using independent samples *t*-test, Mann-Whitney U test, and Chi-square test where appropriate. Spearman’s coefficient and Pearson’s correlation were calculated as suitable to determine the correlation between the biochemical parameters. *P*-values of less than 0.05 were considered statistically significant. The quantitative data were shown as mean±standard deviation (SD) and median (min–max) as suitable.

## Results

Organs’ involvement and baseline characteristics of the scleroderma patients and controls are summarized in Tables 1 and 2. The most involved organs in the patient group were peripheral vasculature (100%) and skin (98.40%) and the least involved ones were heart (1.70%) and kidneys (3.33%). Phosphorus concentrations and ALP activity were higher in the scleroderma group than the control: (3.72±0.30 vs. 3.61±0.13, mg/dl; *P*=0.020) and (218.88±67.20 vs. 154.10±22.70, IU/L; *P*<0.001), respectively.

**Table 1:** Demographic characteristics and biochemical parameters in the studied groups

**Variables**	**Scleroderma (n=60)**	**Control (n=30)**	***P* value**
Age (yr) (mean ± SD)	46.15±9.63	43.13±7.52	0.137*
Sex [male/female (n, %)]	7 (11.70)/53 (88.30)	5 (16.70) 25 (83.30)	0.525**
Phosphorus (mg/dl) (mean ± SD)	3.72±0.30	3.61±0.13	0.020*
Calcium (mg/dl) (mean ± SD)	9.12±0.24	9.21±0.19	0.082*
Alp (IU/l) (mean ± SD)	218.88±67.20	154.10±22.70	<0.001*
AST(IU/l) (mean ± SD)	18.80±3.42	17.40±4.83	0.164*
ALT(IU/l) (mean ± SD)	20.08±3.52	18.06±4.93	0.28*
Urea(mg/dl) (mean ± SD)	28.50±1.90	28.23±2.32	0.563*
Creatinine (mg/dl) [median (min–max)]	0.90 (0.70–1.40)	0.90(0.6–1.30)	0.730***

Note: ALT, Alanine transaminase; AST, Aspartate transaminase; Alp, alkaline phosphatase

* *P-*value based on independent sample t-test;

** *P*-value based on Chi-square test;

*** *P*-value based on Mann-Whitney U test

**Table 2:** Clinical characteristics of the scleroderma patients

Duration of disease (yr) (mean±SD)	6.63±2.73
Anti slc70 positive (n, %)	34. 56.70
Anti-centromere Positive (n, %)	2 (3.30)
Calcinosis Positive (n, %)	14 (23.3)
Organ involvement	(n, %)
Heart	1 (1.70)
Joints	23 (38.40)
Kidneys	2 (3.33)
Lungs	34 (56.70)
Skin	59 (98.40)
Muscles	6 (10)
Gastrointestinal	55 (91.70)
Peripheral vascular	60 (100.00)

Note: Anti scl70, anti-topoisomerase 1

As shown in Table 3, serum Klotho and 25-OH Vit D concentrations were significantly lower in the scleroderma patients than those in the control group: [3.47 (2.30–11.07) vs. 4.28 (2.99–7.88), ng/mL; *P*<0.001] and [15.01±4.71 vs. 27.23±8.66, ng/mL; *P*<0.001], respectively. Mean serum levels of iPTH in the patients with scleroderma was higher than those in the healthy controls [17.83±8.52 vs. 12.06±2.44, pg/ml; *P*<0.001]. There was no significant difference in FGF-23 levels between the patients and controls [32.44 (27.19–68.07) vs. 31.85(20.00–51.69), pg/ml; *P*=0.202). In the sub-group analysis, based on diffused and limited forms of the disease, no significant differences were found in serum Klotho, FGF-23, vitamin D, and iPTH levels (Table 4).

**Table 3:** Klotho, FGF-23, vitamin D and iPTH levels in the scleroderma and control groups

**Variable**	**Scleroderma (n=60)**	**Control (n=30)**	**P value**
Klotho (ng/ml) [median (min–max)]	3.47 (2.30–11.07)	4.28 (2.99–7.88)	<0.001*
FGF-23 (pg/ml) [median (min–max)]	32.44 (27.19–68.07)	31.85 (20.00–51.69)	0.202*
25-OH D (ng/ml) (mean ± SD)	15.01±4.71	27.23±8.66	<0.001**
iPTH (pg/ml) (mean ± SD)	17.83±8.52	12.06±2.44	<0.001**

Note: FGF-23, fibroblast growth factor 23; 25-OH D, 25-hydroxy vitamin D; iPTH, intact parathyroid hormone

* *P*-value based on Mann-Whitney U test;

** *P*-value based on independent sample t-test

**Table 4:** Klotho, FGF-23, vitamin D and iPTH levels in the limited and diffused forms of scleroderma

**Variable**	**Limited (n=30)**	**Diffused (n=30)**	**P-value**
Klotho (ng/ml) [median (min–max)]	3.58(2.50–8.73)	3.43(2.30–11.07)	0.352*
FGF-23(pg/ml) [median (min–max)]	32.33(28.09–57.04)	32.49(27.19–68.07)	0.451*
25-OH D (ng/ml) (mean ± SD)	14.50±4.73	15.54±4.71	0.398**
iPTH (pg/ml) (mean ± SD)	18.08±8.45	17.58±8.72	0.823**

Note: FGF-23, fibroblast growth factor 23; 25-OH D, 25-hydroxy vitamin D; iPTH, intact parathyroid hormone

* *P*-value based on Mann-Whitney U test;

** *P*-value based on independent samples t-test

### Correlations of Klotho, FGF-23, vitamin D, and iPTH

Table 5 summarizes the relationship between serum levels of Klotho, 25-OH Vit D, and iPTH in the two studied groups. In the patient group, there was a significantly negative correlation between iPTH and 25-OH Vit D (*P*<0.001, r=−0.531), but in the control group, there was no significant correlation between the variables.

**Table 5:** Correlation between main factors in the scleroderma and control groups

**Groups**	Klotho and 25-OH D*		Klotho and iPTH*		iPTH and 25-OH D**	
	***r***	***P***	***r***	***P***	***R***	***P***
Scleroderma	−0.145	0.270	0.136	0.301	−0.531	<0.001
Control	0.278	0.137	0.097	0.610	−0.150	0.430

Note: FGF-f23: fibroblast growth factor 23; 25-OH D, 25-hydroxy vitamin D; iPTH: intact parathyroid hormone

* Spearman’s correlation;

** Pearson’s correlation

## Discussion

Scleroderma is a multisystem disease with a variable clinical course and poor prognosis. Microvascular damage in scleroderma provokes the immune cells to produce autoantibodies as well as pro-inflammatory and pro-fibrotic cytokines and chemokines (14). Several epidemiological studies have shown that vitamin D deficiency could contribute to the risk of autoimmune diseases such as scleroderma (6). Vitamin D receptor (VDR) is expressed in multiple immune cell types such as monocytes, dendritic cells, and activated T cells. It can be potentially relevant to the susceptibility and development of autoimmune diseases (15–18). In the present study, we evaluated some of the main factors involved in calcium and phosphate homeostasis including serum FGF-23, Klotho, vitamin D, and iPTH levels in the scleroderma patients compared with the healthy individuals.

Vitamin D deficiency could play a significant role in the reduction of bone mineral density and total mineral content. The result showed that the serum concentration of vitamin D was lower in the patients than controls. Lower levels of vitamin D in these patients have been confirmed by previous studies reported reducing vitamin D in the majority of scleroderma patients (7, 19). Thickening of skin in scleroderma patients (20) may lead to the decline in the production of vitamin D in the skin after being exposed to UV light. Additionally, gastrointestinal involvement in this disease can reduce the absorption of vitamin D (7, 19).

Klotho is an anti-aging single-pass transmembrane protein predominantly produced in the kidney, with shedding of the amino-terminal extracellular domain into the systemic circulation as s-Klotho (21). Circulating levels of s-Klotho are decreased with age, and the defect in the Klotho gene is associated with the increased risk of age-related diseases.

According to the results, scleroderma patients had significantly lower serum Klotho concentration than the controls. Systemic inflammation and increased inflammatory markers such as IL-4 and IL-13 are associated with scleroderma (22, 23). Since high inflammatory markers can reduce renal Klotho expression (24). Low levels of Klotho in the scleroderma patients in our study might be due to the systemic inflammation and increased inflammatory markers in the diseases. The bone-derived hormone, FGF-23, and its co-receptor, Klotho, depict a novel endocrine axis regulating mineral metabolism in health and disease states. Binding of FGF-23 to FGF receptors on target cells and its subsequent action require the presence of its co-receptor, Klotho (25, 26). FGF-23 downregulates the nephritic proximal tubular reabsorption of phosphate and impedes the renal synthesis of the most active form of vitamin D, 1.25-dihydroxyvitamin D-3 (1.25-(OH)_2_ D3), through suppressing renal 1α-hydroxylase (27). Therefore, FGF-23/Klotho signaling represses renal phosphate reabsorption as well as the activation of vitamin D. This signaling can also reduce secretion PTH (28). In our study, although there was no significant difference in FGF-23 levels between the two studied groups that were in agreement with the study (29), higher iPTH and phosphate levels in scleroderma group might be due to lower Klotho levels in these patients compared with the control group.

In the present study, there was no significant difference in calcium levels between the two studied groups, which might be due to increased PTH and decreased vitamin D in scleroderma patients compared with the healthy controls. In a long run, this mechanism might decrease the bone density of scleroderma patients.

Our data showed a significantly negative correlation between iPTH and 25-OH Vit D in the patient group. One of the important functions of PTH is the conversion of 25-hydroxyvitamin D into 1, 25-(OH)_2_ D3 (its most active metabolite) by the activation of the enzyme 1α-hydroxylase in the proximal tubules of the kidney. Although not well elucidated, 1.25-(OH)_2_ D3 appears to exert an inhibitory effect on the parathyroid gland (30). The results of the present study also demonstrated no differences in serum FGF-23, Klotho, 25-OH Vit D, and iPTH levels between the diffused and limited forms of scleroderma. Therefore, these measured factors are not sensitive enough to predict the severity and/or distinguish between the two forms of the disease.

Some weaknesses of our study were no measurement of bone density and the possible effects of consumed drugs in the scleroderma patients on Klotho, vitamin D, iPTH, as well as FGF-23 levels.

## Conclusion

Serum Klotho and 25-OH Vit D levels were declined, but iPTH serum levels were increased in scleroderma disease. These factors may have a role in the pathogenesis of scleroderma considered helpful markers in the diagnosis of the disease, without distinguishing the diffused form and the limited form of this disease.

## Ethical considerations

The ethics committee at the Tabriz University of Medical Sciences reviewed and approved the present study, in compliance with the Declaration of Helsinki. Informed consent was obtained from all participants.
